# A Pipeline for Automating Emergency Medicine Documentation Using LLMs with Retrieval-Augmented Text Generation

**DOI:** 10.1080/08839514.2025.2519169

**Published:** 2025-06-18

**Authors:** Denis Moser, Matthias Bender, Murat Sariyar

**Affiliations:** Department Medical Informatics, Bern University of Applied Sciences, Biel/Bienne, Switzerland

## Abstract

Accurate and efficient documentation of patient information is vital in emergency healthcare settings. Traditional manual documentation methods are often time-consuming and prone to errors, potentially affecting patient outcomes. Large Language Models (LLMs) offer a promising solution to enhance medical communication systems; however, their clinical deployment, particularly in non-English languages such as German, presents challenges related to content accuracy, clinical relevance, and data privacy. This study addresses these challenges by developing and evaluating an automated pipeline for emergency medical documentation in German. The research objectives include (1) generating synthetic dialogues with known ground truth data to create controlled datasets for evaluating NLP performance and (2) designing an innovative pipeline to retrieve essential clinical information from these dialogues. A subset of 100 anonymized patient records from the MIMIC-IV-ED dataset was selected, ensuring diversity in demographics, chief complaints, and conditions. A Retrieval-Augmented Generation (RAG) system extracted key nominal and numerical features using chunking, embedding, and dynamic prompts. Evaluation metrics included precision, recall, F1-score, and sentiment analysis. Initial results demonstrated high extraction accuracy, particularly in medication data (F1-scores: 86.21%–100%), though performance declined in nuanced clinical language, requiring further refinement for real-world emergency settings.

## Introduction

In healthcare settings, especially in high-pressure environments like emergency care and rescue services, the ability to document and relay patient information swiftly and accurately is critical. Comprehensive and precise documentation plays a key role in clinical decision-making, ensuring continuity of care, and ultimately improving patient outcomes. However, traditional manual documentation methods often prove to be time-consuming and prone to inaccuracies, particularly when relying on retrospective processes. These inefficiencies can significantly impact the quality of patient care (Endsley 2011). The integration of advanced technologies, such as large language models (LLMs) and hands-free platforms, into medical communication processes holds promise in addressing these challenges. By enhancing real-time documentation, these technologies support clinicians in maintaining accurate records without the usual time delays, improving both the efficiency and reliability of patient information exchange.

The state of the art in hands-free electronic documentation for emergency care is evolving rapidly, with several promising technologies emerging to address the critical need for efficient and accurate documentation (Falcetta et al. 2023). Automated systems, such as the Automated Sensing Clinical Documentation system (Bloos et al. 2020) and platforms leveraging hands-free technology (like smart glasses), have shown feasibility in real-world settings, although they face challenges related to noise resilience and usability in chaotic medical environments (Zhang et al. 2022). Most of the systems rely on advanced automatic speech recognition and natural language processing (NLP) to capture medical information in real-time, reducing the burden on clinicians and minimizing errors (Woo et al. 2021). While digital scribes demonstrate increased efficiency and ease of use, especially in patient-centered communication, many of these systems still require further optimization and large-scale validation to become fully integrated into clinical workflows (Wang et al. 2021). Nonetheless, these innovations signal a shift toward more seamless, real-time documentation processes that support emergency care providers under intense conditions.

Nowadays, cloud – based large language models achieve state-of-the-art performance on medical information-extraction tasks (García-Barragán et al. 2024; Yang et al. 2024). However, transmitting protected health information to third-party servers poses serious privacy risks in real-world medical applications. In contrast, deploying LLMs on-premises preserves patient confidentiality. Although local LLMs – especially smaller, off-the-shelf open-source variants – often underperform relative to their cloud-hosted counterparts, they can still deliver acceptable results when properly managed. Two strategies that help close this performance gap are fine-tuning on domain-specific examples and in-context learning via prompt engineering. In particular, carefully designed prompts can reduce hallucinations and improve robustness, even for out-of-the-box local models (Alkhalaf et al. 2024).

A further challenge for on-premises deployments is the limited context window of local LLMs, which caps how much text can be processed in a single inference pass. Complete Emergency Medical Services (EMS) dialogue transcripts – whose lengths and participant counts vary widely – might exceed this limit, forcing either the deployment of larger models or iterative processing of the text, both of which drive up latency and compute requirements. To mitigate this, we propose that a lightweight semantic-retrieval step can pre-filter transcripts for the most clinically relevant passages before costly LLM inference. In other words, the model receives a curated subset of candidate passages – omitting irrelevant content – thereby, in theory, preserving the extraction performance of local LLMs while keeping hardware demands stable regardless of transcript length.

Information-extraction (IE) pipelines require representative data for both model development and evaluation, yet no publicly available corpus of German EMS dialogue transcripts exists. The only related resource, RescueSpeech, comprises 2 h of firefighter-simulation audio, is domain-mismatched, and still requires speech-to-text conversion (Sagar et al. 2023). Recording and manually annotating authentic EMS dialogues entails complex privacy-compliance procedures and substantial annotation effort – burdens that become prohibitive when project resources are limited. As an alternative, synthetic dialogue generation via LLMs offers a scalable, privacy-compliant path.

Prior work on medical dialogue generation stem mainly from China. Platforms such as Chunyu Doctor provide millions of unlabeled dialogues that underpin MedDialog (Zeng et al. 2020), KaMed (Li et al. 2021), ReMeDi-large (Yan et al. 2022), and MedDG (Liu et al. 2022). Building on these datasets, systems like NoteChat generate dialogues from medical notes (Wang et al. 2024), while MedKP enriches ChatGLM3-6B with external knowledge to boost performance on KaMed and MedDG (Wu et al. 2024); reasoning-focused methods such as BP4ER further refine generation quality (He et al. 2024). In the English-speaking world, resources are scarcer: AskADoctor data and 10 000 iCliniq dialogues enabled ChatDoctor (Li et al. 2023). Together, these examples highlight the central role of domain-specific dialogue corpora and underscore the current gap for medical language processing – prompting us to generate synthetic German medical emergency service dialogues primarily as a testbed for developing and evaluating our LLM-based information-extraction pipeline.

Generating useful medical dialogues requires the integration of clinically coherent information and the availability of ground-truth annotations to support downstream information extraction benchmarks. A practical method to fulfill both requirements involves incorporating structured clinical data from available real datasets into the prompt context for language model-based generation. This approach ensures semantic and factual consistency within the dialogue and simultaneously provides gold-standard labels that can be leveraged for evaluation tasks. However, data use agreements often strictly prohibit transmitting raw or potentially identifiable patient information to external cloud-based services, thereby restricting access to high-capacity, proprietary language models when local or open-source LLMs prove insufficient. This regulatory constraint necessitates a preprocessing step wherein records are transformed into fully synthetic, de-identified datasets that preserve clinical plausibility while ensuring privacy compliance prior to their use in data-driven dialogue generation

To address privacy constraints, data scarcity, and context window limitations in LLMs, we propose a three-part framework tailored to the complexities of German emergency service dialogues. (1) A retrieval-augmented generation (RAG) pipeline extracts clinically relevant information from noisy, unstructured transcripts, where medical details are often scattered and interwoven with non-clinical content; we hypothesize that sufficiently large pretrained models can perform this task without fine-tuning, leveraging their inherent medical knowledge. (2) A synthetic dialogue generator produces annotated German emergency conversations from structured MIMIC-IV data, embedding realistic medical content alongside distractions such as irrelevant details, redundancies, and terminological variation to simulate real-world conditions. (3) An adapted Post-Randomization Method (PRAM) converts MIMIC-IV records into fully synthetic, de-identified inputs that comply with licensing restrictions while preserving clinical realism. Unlike prior work – e.g., Heilmeyer et al., who lacked controlled ground truth (Heilmeyer et al. 2024), or Wang et al., who focused on static texts (Wang et al. 2024) – our pipeline enables scalable, automated evaluation of information extraction in dynamic, multilingual emergency scenarios, providing a privacy-preserving testbed for LLM-based clinical documentation.

## Methods

### Data basis: MIMIC-IV dataset

MIMIC-IV, hosted on PhysioNet and provided by the Massachusetts Institute of Technology, is a publicly available database containing de-identified patient data from Beth Israel Deaconess Medical Center in Boston. It is intended for research in healthcare and biomedical fields, offering a wide range of clinical information (Johnson et al. 2023). A specific subset, MIMIC-IV-ED, focuses on the Emergency Department (ED), providing detailed records of patient encounters, including diagnoses, vital signs, and treatments. This dataset was utilized in our study to generate an annotated dialogue text dataset for system design and evaluation.

We utilize structured data from randomly sampled 100 patients as the baseline for our system, serving two principal objectives. First, this dataset facilitates the generation of dialogues for emergency scenarios based on consistent patient characteristics. Second, it provides a gold standard for evaluating the efficacy of our NLP pipeline in text extraction. To address privacy concerns and comply with data usage terms, we additionally anonymize the baseline data before processing it, thereby eliminating any direct association with the original sources. Consequently, our analysis is based solely on this synthetic data (see next subsection).

Table 1 delineates the features employed in this study to ensure a representative spectrum of patient profiles. These features were identified following the storage of the MIMIC-IV data in our MongoDB database. Initially, we determined the relevant features by evaluating the data’s alignment with our research objectives. The selection of specific tables from the MIMIC-IV database – namely edstays, diagnosis, medrecon, pyxis, triage, and vitalsign – was guided by their capacity to capture comprehensive patient profiles in accordance with the German DIVI and MIND emergency protocols (Messelken et al. 2011). This targeted selection ensures that the dataset encompasses the critical variables necessary for robust and representative patient profiling.

**Table 1.** Overview of the MIMIC-IV data.

ED collection (table)	Feature (key columns)	Description	Example values	Injected directly into prompt?	Ground truth
edstays, diagnosis, medrecon, pyxis, triage, vitalsign	stay_id	Unique identifier for each ED visit.	“34928444”	No. Only used for data organization.	No
edstays	arrival_transport	Mode of patient arrival.	“AMBULANCE,” “WALK IN”	No. Only used for filtering out “walk in” cases.	No
triage	acuity	Severity level of the patient’s condition.	”2.0000”	No. Only for choosing prompt templates.	No
edstays	gender	Patient’s gender.	“F,” “M”	Yes	No
diagnosis	icd_title	Diagnosis description (ICD code title).	“Altered mental status, unspecified”	Yes	Yes
medrecon	name	Medication name, patient reported.	“Vitamins - D Derivatives”	Yes	Yes
pyxis	name	Medication name, administered	“PredniSONE 20mg TAB”	Yes	Yes
triage, vitalsign	temperature	Patient’s body temperature.	”97.8000”	Yes	Yes
triage, vitalsign	heartrate	Patient’s heart rate.	”78.0000”	Yes	Yes
triage, vitalsign	resprate	Patient’s respiratory rate.	”16.0000”	Yes	Yes
triage, vitalsign	o2sat	Oxygen saturation level.	”100.0000”	Yes	Yes

The edstays table offers fundamental details about patient admissions, including the mode of arrival and transport. Complementing this, the diagnosis table records patient diagnoses coded with ICD titles. The medrecon table includes data on medications reported by patients prior to their ED visit, which is crucial for comprehending the patient’s medication history and ensuring continuity of care. In contrast, the pyxis table details the medications administered during the visit. By comparing this data with the pre-visit medications recorded in medrecon, discrepancies can be identified, ensuring that the medication administration is accurately reflected and aligns with actual practices. Furthermore, the triage table documents patient acuity levels and initial vital signs recorded upon arrival. This information is essential for assessing the severity of the patient’s condition and prioritizing treatment based on urgency. In addition, the vitals table provides detailed measurements of vital signs throughout the ED visit. Key parameters, such as blood pressure, temperature, heart rate, respiratory rate, and oxygen saturation (O2 saturation), are critical for monitoring the patient’s health status and evaluating their response to treatment.

Although not all protocol fields could be mapped to the ED data, the identified features provided a sufficient foundation for generating dialogues and exploring the quality of our information extraction NLP pipeline in emergency service scenarios.

#### Ethical approval statement

This study did not involve human participants, human data, or human tissue. Therefore, ethical approval was not required. No approval from an ethics committee was necessary. Consequently, the study does not fall under the scope of the Declaration of Helsinki.

### Synthetic data generation using PRAM

The MIMIC-IV dataset we obtained is anonymized to protect patient identities, but it remains subject to strict data-sharing restrictions that prohibit dissemination to third parties, such as OpenAI. To comply with these restrictions, we swapped feature values according to the PRAM method (Domingo-Ferrer and Soria-Comas 2018). Compared to data swapping, PRAM offers a significant advantage: it is grounded in statistical principles rather than being purely mechanical. This allows for the application of comprehensive statistical modeling and inference techniques. In this context, our primary focus is on achieving non-mechanical perturbation of the features, ultimately rendering it entirely synthetic.

The PRAM implementation used in our study is based on the sdcMicro package from R. We determine the transition matrix probabilities P such that there is a positive probability of a swap between the i-th and j-th category values ($p_{\mathrm{ij}}>0,i\neq j$), while ensuring that $p_{\mathrm{ii}}=0$. This approach is taken to generate fully synthetic data. Gender is preserved in the dataset to avoid inconsistencies, such as mismatches between gender-specific diagnoses or medications, particularly in the context of dialogue generation.

To preserve data coherence and thereby data utility as much as possible, original values are swapped only with similar values. For numerical data, like blood pressure readings, we can directly swap values using the PRAM function provided by the sdcMicro package. This method automatically ensures that the swapped values remain within a plausible range for the attribute. Nominal data (like diagnoses or drug names), which are represented as text strings, presented a challenge. Automated swapping of these values using PRAM was not directly feasible because it requires a transition matrix that connects similar words or categories, which is difficult to generate automatically for text data. To construct the transition matrices in this case, we randomly sampled 1,000 unique patient cases, enabling the exchange of semantically similar values.

To illustrate the principle of utility-based PRAM using text data, we focus here on the diagnostic descriptions. We include only unique diagnoses of the previously sampled 1000 patient cases into a sub-sample dedicated for clustering, to prevent data skewing that could result from multiple identical entries. The distinct diagnoses are then categorized into semantically similar groups based on the similarities of their corresponding word embeddings (see below). For this purpose, we employ the “bionlp/bluebert_pubmed_mimic_uncased_L-12_H-768_A-12” model (Peng, Yan, and Lu 2019), which is specifically trained on biomedical texts, allowing for a more accurate representation of word meanings within this context. Initially, the word embeddings are 768-dimensional. To enhance data manageability, the dimensionality of these embeddings is reduced using Principal Component Analysis. For instance, in the context of diagnoses, the dimensions are reduced to 65. This reduction simplifies the data while retaining as much meaningful information as possible.

The similarities of the dimensionally reduced word embeddings are calculated as follows: First, the word embeddings are L2-normalized, ensuring that each embedding vector has a unit length. This normalization step transforms the L2 distance into a linearly affine version of the cosine distance, which is more appropriate for semantic comparisons. Following normalization, the embeddings are grouped using constrained k-means clustering, implemented via the “k-means-constrained” library (Su, Kogan, and Nicholas 2010). Unlike standard k-means clustering, which can produce clusters of varying sizes, this method allows for the specification of cluster sizes. In this case, the clustering is constrained to produce 100 clusters of equal size. Each of the diagnostic string is then assigned a cluster number based on its grouping. Within each cluster, outliers are identified by calculating the cosine distance of each string from the cluster center. Strings exceeding three standard deviations above the mean cosine distance within their cluster (>3σ) are flagged as outliers. To maintain cluster integrity, the single most extreme outlier in each cluster is removed, not more, ensuring the clusters remain sufficiently large.

**Figure 1.**
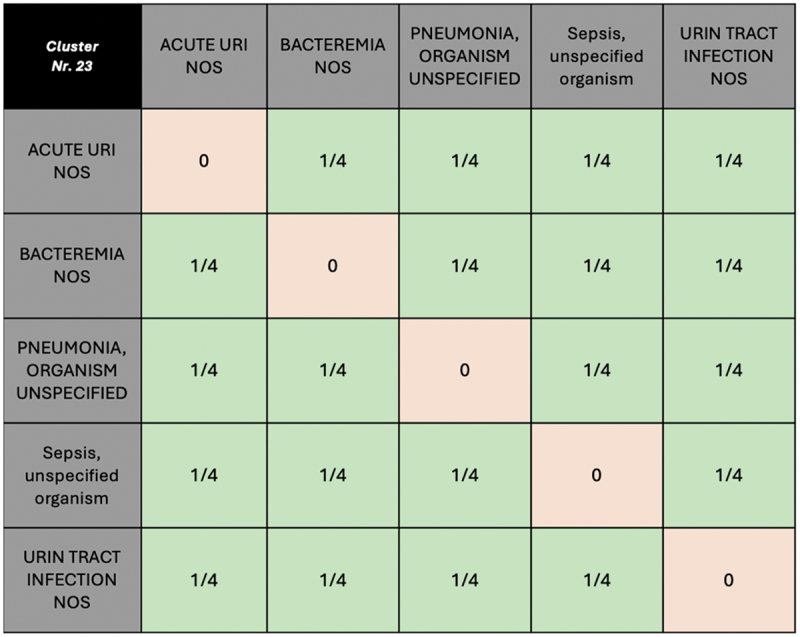
A conceptual depiction of a transition matrix for diagnoses falling into cluster nr. 23. Diagonal cells are zero (no self-transition), while off-diagonal cells have equal probabilities (in this case 1/4 or 25%) for transitioning to other complaints during the PRAM process.

The clustering results are subsequently mapped back to the original 1000 samples, with each sample assigned a corresponding cluster number based on the proximity to the nearest cluster center. During PRAM for each sample, the cluster number is picked and used to build a transition matrix with all string values within given cluster, including the original value. For each transition matrix, a uniform distribution was applied to all non-original values, while the original value is assigned a probability of 0, enforcing replacement of the old with the other values in given cluster (see Figure 1).

The resulting modified values were then stored in the MongoDB database as the final synthetic dataset, for the generation of 100 dialogues.

### Dialogue generation pipeline

Based on the synthetic MIMIC dataset, we generate 100 dialogue texts within an emergency context, aiming to simulate verbal interactions between healthcare professionals and patients. To achieve this, we develop a two-stage pipeline for automated dialogue generation: an initial local generation of English dialogues followed by refinement and translation into German via OpenAI’s API services. In the first stage, we employ the “Zephyr-7b-beta” model from the Hugging Face transformers library, which offers performance comparable to larger models like Llama-2-70B-Chat. The model is configured with a maximum token length of 1500 and a temperature setting of 0.7 to introduce variability in the dialogues (Peeperkorn et al. 2024). In the second stage, we refine and translate the initially generated English dialogues into German using OpenAI’s GPT-4 Turbo model with again a temperature setting of 0.7. The primary objectives here are to enhance naturalness, fluency, and extend the dialogue length in token size, while keeping relevant clinical information intact for later evaluation.

**Figure 2.**
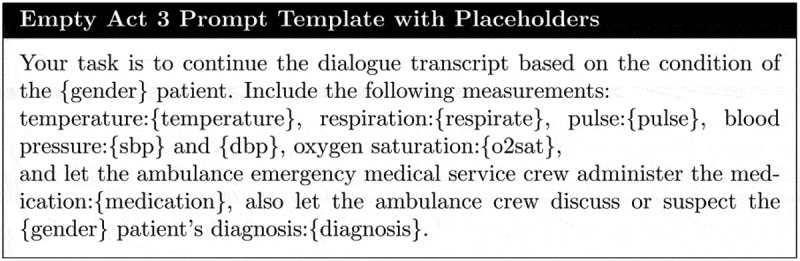
One of the prompt templates used for dialogue generation containing placeholders to be injected with MIMIC-IV based data.

There are two primary reasons for initiating the first stage: (i) to further distance the process from the synthetic MIMIC-IV dataset before transitioning to cloud-based models accessed via the OpenAI API services, and (ii) to generate dialogues that minimize challenges in text extraction. The initial dialogue generation process is divided into four sections to ensure a coherent narrative structure: (1) initial ambulance interaction, (2) triage and initial vital sign measurements, (3) administration of medication and subsequent vital sign measurements, and (4) hospital arrival. For each phase, we design a prompt template that instructs the language model on the specific events, the actions of healthcare professionals (such as medical procedures), and the synthetic data features to be integrated (Figure 2 shows a template for the phase (3)).

**Figure 3.**
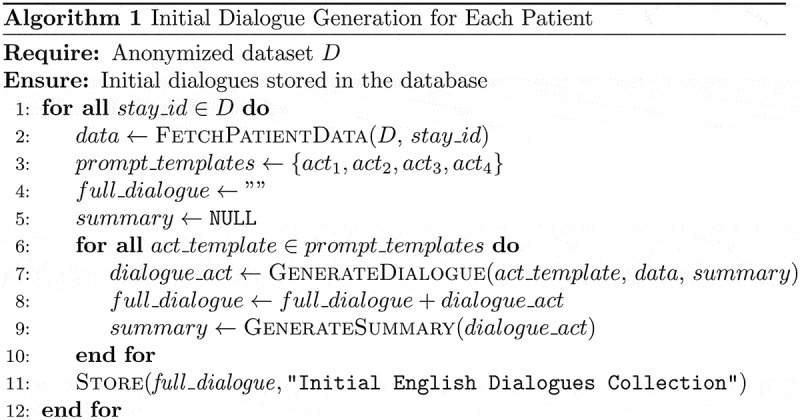
Conceptual pseudocode illustrating the iterative process of initial dialogue generation in English, utilizing the local Zephyr-7b-beta model.

This initial process can be summarized and conceptually illustrated by the following pseudocode, which captures the underlying implementation concept (Figure 3).

**Figure 4.**
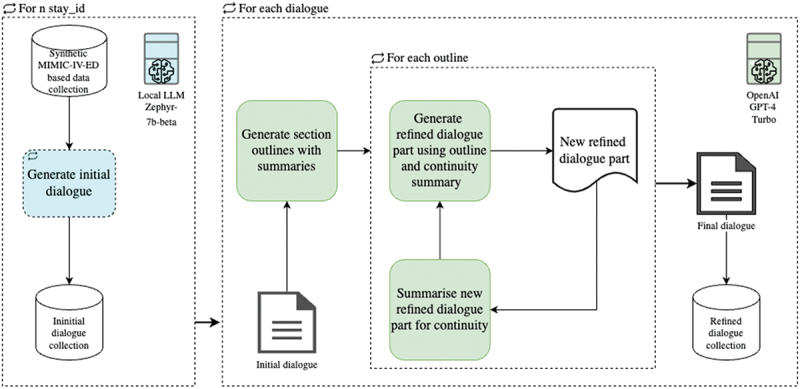
Hierarchical expansion process for dialogue generation. This flowchart illustrates the multi-step process of generating and refining dialogues using a combination of local and cloud-based LLMs. Starting with synthetic data collection, an initial dialogue is generated and then iteratively refined through outlining, summarization, and continuity checks. The process culminates in the creation of a high-quality final dialogue, leveraging advanced LLMs for optimal results.

For generating the extended German dialogues, we use the initial dialogues as a foundational basis. Although models like GPT-4-turbo from OpenAI have large context windows (e.g., accepting up to 128,000 tokens), they are still limited by a maximum output length. To circumvent the constraints of context length, we employ a hierarchical expansion method. Unlike overlapping chunking, this approach significantly reduces the occurrence of contextual drift and incoherent output dialogues (Nuttal 2023). The hierarchical strategy facilitates detailed and coherent dialogue generation, ensuring that each segment is meticulously refined and seamlessly integrated into the overarching narrative. This process is illustrated in Figure 4. For each generated initial dialogue, the system produces summaries of individual sections. These summaries serve as guides for the subsequent refinement process. Each section’s outline is used to generate a refined dialogue part, which is then summarized and prefixed to the prompt template for the next section, ensuring continuity throughout the dialogue. The refined segments are then assembled to create the final dialogue.

The templates for generating refined dialogues build significantly on those used for the initial dialogues, with three key enhancements aimed at producing more natural and realistic exchanges. First, each refined dialogue begins with a section summary that encapsulates the content of the initial dialogues. Second, from section two onward, cumulative summaries of the preceding refined sections are included, enriching the context. Placeholders are defined for both summary types. Third, the prompt includes detailed delivery instructions – such as tone, language requirements (German), and context sensitivity. A crucial feature is the “Current Events” block, which summarizes all preceding medical facts, allowing the model to adjust tone and urgency – from life-threatening events like cardiac arrest to minor issues like dizziness – via the clause: “Ensure that the dialogues are authentic, appropriate for an emergency medical response in German, and grounded in the Current Events.” The prompt also defines clear register boundaries: “Medical professionals should communicate with each other in specialist terms while speaking to the patient in plain, courteous language.” Additionally, it conditions the patient’s responses on clinical status, e.g., “Adjust the patient’s responses to their medical condition – for example, if confused or critically ill, they should speak accordingly.” These layered instructions ensure that the generated dialogues are situationally appropriate and linguistically authentic. The final dialogue sections are compiled and stored in the MongoDB database.

**Figure 5.**
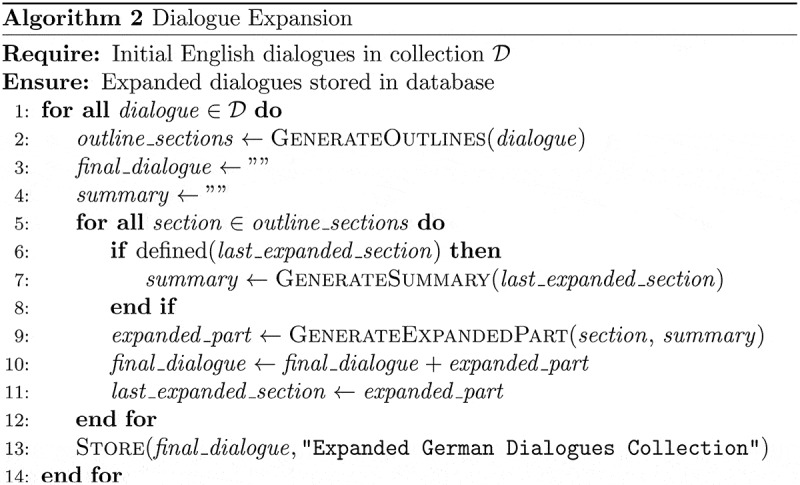
Pseudocode representation of the iterative refinement, translation into German, and expansion process for dialogue generation, utilizing the OpenAI GPT-4 Turbo API.

The iterative process for refining and expanding the dialogues is conceptually represented in Figure 5 as pseudocode, illustrating the key steps from generating section outlines to storing the complete refined dialogues.

### RAG for text extraction

To extract the desired information in accordance with emergency medical protocols, several methods can be employed. Traditional techniques, such as named entity recognition (NER), often encounter challenges in capturing the necessary contextual nuances, resulting in incomplete or inaccurate outcomes (Cho and Lee 2019). This is particularly problematic in cases where the information to be extracted is highly heterogeneous, as these methods tend to miss subtle but critical details that are context-dependent (Landolsi, Hlaoua, and Romdhane 2024). In contrast, RAG offers a more promising approach and combines two key steps to improve how information is processed and generated. First, it retrieves relevant data or documents from a database or vector store, which helps it understand the context better. Then, it uses this information to generate more accurate and relevant responses or outputs. Essentially, RAG finds the right information first and then uses it to create a better answer, making it particularly useful for tasks where context and detail are important.

When deploying local LLMs, particularly in scenarios where the sensitivity of patient data precludes the use of cloud-based models, it is crucial to implement efficient text extraction techniques that prioritize the most relevant sections of the text. Due to the context length limitations inherent in LLMs, processing entire documents simultaneously is often impractical. Therefore, a sophisticated chunking strategy must be developed in conjunction with RAG methods to optimize processing efficiency and ensure accurate outcomes. Our RAG approach involves four key steps:

1. **Chunking**: We divide each dialogue text into overlapping chunks of 200 characters, which are then embedded and stored in a vector database.
2. **Prompt Template Creation**: We design a dynamic prompt template with placeholders (see Figure 6) that can be adapted to various extraction targets. This template allows for the specification of granular queries for retrieval from the indexed chunks in the vector store.

**Figure 6.**
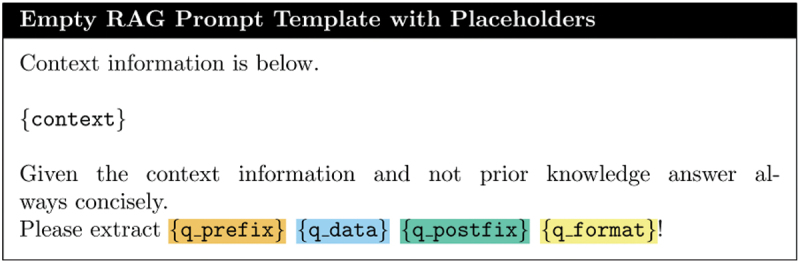
Empty RAG prompt template for answer generation with placeholders.
3. **Chunk Retrieval**: Relevant chunks are retrieved from the vector store based on the top k similarity scores with the query data q_data (see Figure 7 for a filled-in prompt). To optimize retrieval accuracy, only q_data is utilized, minimizing the likelihood of retrieving less relevant chunks.

**Figure 7.**
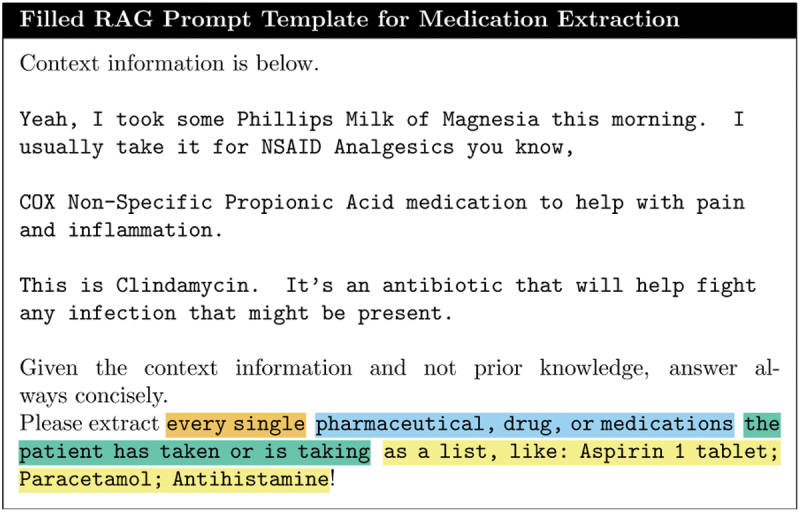
Filled RAG prompt template delivered to LLM, with abbreviated context (chunks retrieved from dialogue). In this case we want to extract “Phillips Milk of Magnesia”.
4. **Answer Generation**: The complete prompt, including the retrieved context and placeholders, is then provided to the multilingual MaziyarPanahi/Llama-3-8B-Instruct-v0.8 model (temperature: 0.2). The model processes the prompt and returns the extracted information as a list.

This method ensures that the LLM can efficiently process and extract relevant information from text, while adhering to privacy constraints and maximizing accuracy.

In step 1, we use a loop where each dialogue is divided into segments of 200 characters, with a 40-character overlap. Utilizing the LangChain library, these segments are embedded using the multilingual embedder model (intfloat/multilingual-e5-large-instruct) and indexed in a FAISS (Facebook AI Similarity Search) vector store (Ke et al. 2024). FAISS allows similarity search and is configured to retrieve the top k = 12 most relevant segments upon receiving the target (q_data). Before delivering the prompt to the LLM, FAISS retrieves relevant segments from its vector store based on the specified q_data value (e.g., pharmaceuticals, drugs, and medications). These retrieved text chunks form the context part in the prompt.

It is important to recognize that German dialogues are often less effectively processed by pre-trained models compared to English texts, primarily due to the larger English corpora used during multilingual model training. This results in performance gaps when handling German medical dialogues. To address this, we translate the refined German dialogues back into English to enhance the local LLM’s extraction performance. Even when using the same multilingual LLM, translating before extraction yields better results, as translation is a generative task that preserves context more effectively. By translating first, we effectively bridge into the high-resource English domain, where the model’s extraction capabilities are more accurate and reliable. While fine-tuning on sufficient German data could mitigate this gap, limited access to German medical texts – due to strict data privacy regulations – remains a significant constraint.

We select the MaziyarPanahi/Llama-3-8B-Instruct-v0.8 model because it ranked in the top 5 on the Hugging Face open LLM leaderboard for models with 7–8 billion parameters, aligning well with our hardware capabilities. We run the model on a system with an Intel 13th Gen Core i9-13900K CPU (32 cores, up to 5.8 GHz), 128 GB DDR5 RAM (5200 MHz), and two NVIDIA RTX 4500 Ada GPUs with 24 GB memory. Despite the limitations of LLMs in processing German text for our extraction task, we continue to use the multilingual-e5-large-instruct model. Although German texts are translated into English prior to entering the RAG, this model was initially chosen for its support of 94 languages and robust retrieval capabilities. Translation is used to leverage the richer ecosystem of English-language models, which benefit from broader pretraining and more mature domain-specific tools. To support future multilingual applications, we retain this model as a foundation for cross-lingual development.

### Evaluation

Our evaluation comprises three components: (1) assessing the quality of data generation, (2) evaluating the RAG-based extraction of relevant information, and (3) a manual review of dialogue naturalness by an emergency medical expert with 23 years of experience and over 40,000 processed incidents, using self-created dialogues from real-world scenarios for comparison. The quality assessment is conducted through manual review by an emergency medical technician, complemented by sentiment analysis. It is documented that LLMs tend to generate text with a less negative or toxic sentiment (Deshpande et al. 2023; Meade, Poole-Dayan, and Reddy 2022), for example, due to training on filtered datasets designed to mitigate such responses (Bender et al. 2021). This approach may introduce inaccuracies in scenarios that require a specific emotional tone, such as those typical of emergency service communications. Such a bias may result in the exclusion of critical clinical information, such as suicidal or homicidal ideation, or severe diagnoses, particularly if these details were present in the ground truth but not included in the generated dialogues by the LLM.

#### Dialogue generation evaluation

To assess the quality of the generated dialogues, we employ two complementary direct evaluation methods. First, we evaluate the potential sentiment drift introduced during translation and refinement. Sentence-level sentiment analysis is applied to both the initial English and the refined German dialogues using the distilbert-base-multilingual-cased-sentiments-student model, which classifies each sentence as negative, neutral, or positive. For each dialogue, we compute the proportion of sentences labeled as positive. To test whether the refined German dialogues exhibit a systematic increase in positivity – potentially reflecting sentiment bias introduced by LLM detoxification mechanisms – we perform a one-sided Wilcoxon signed-rank test comparing the positive-sentence proportions of paired English and German dialogues. The null hypothesis states that the proportion of positive sentences in the refined version is greater than or equal to that in the original; the p-value is computed based on the signed rank of the paired differences.

Second, a structured dual-judge evaluation is conducted on a subset of 19 refined German dialogues. Each dialogue is independently rated by a medical domain expert and an LLM-based judge (OpenAI’s o3-mini) across eight dimensions: Naturalness of Language, Persona Consistency, Scenario Plausibility, Turn-taking & Interaction Flow, Context Awareness, Emotional Believability, Factual Accuracy, and Engagement. Ratings are assigned on a three-point Likert scale (1 = poor, 2 = adequate, 3 = excellent), and composite scores are calculated as the mean across all dimensions. Systematic differences between expert and model ratings are analyzed using a paired Wilcoxon signed-rank test. Inter-rater reliability across all ratings is assessed using weighted Cohen’s κ with quadratic weights and asymptotic confidence intervals. An optional free-text field captures qualitative feedback.

#### RAG model performance evaluation

To evaluate the performance of the RAG model, we compare its outputs against the ground truth. We assess whether the responses contain the desired information by employing regular expressions for numerical data and cosine similarity for nominal data. For numerical values, we extract numbers from both the extraction response and the ground truth using re.findall(r’\d+\.?\d*,” text). We then generate a regex pattern for each extracted number and verify its presence in the ground truth using re.search(r”\b{}\b.’format(int(data_value)), answer). To account for minor numerical variations in the dialogues, we also check rounded values and allow deviations of ± 0.5 where appropriate. If a match is found, the number is confirmed as present and processed in the metric calculations accordingly. Given the expected complexity of the dialogues, including abbreviations potentially used among emergency responders, preprocessing is required for nominal data extraction. GPT-4 is employed to standardize terms (e.g., converting “N/V” to “Nausea/Vomiting”) and, when necessary, to separate combined terms into individual components (e.g., “Nausea Vomiting” into “Nausea” and “Vomiting”). Cosine similarity is then applied to the preprocessed data, followed by a visual inspection of the scatter distribution to determine classification thresholds.

To evaluate the effectiveness of the system, we use the following measures: True Positives: Instances where the model accurately identified and extracted relevant clinical information, with the information being mentioned at least once in its response. False Positives: Instances where the model either identified irrelevant information or failed to mention relevant clinical information, even once. False Negatives: Instances where the model failed to identify any relevant clinical information or responded with “No information available.” True Negatives are not included in our evaluation. This is because the dialogue generation pipeline incorporates ground truth into the text, meaning there are no legitimate cases of non-extraction (i.e., there would be no fields in the protocol that should remain unfilled). From these metrics, we calculated Precision, Recall, and F1-Score to quantify the system’s performance. Additionally, 95% confidence intervals (CIs) for each F₁-score are analytically derived using the Delta method, based on the observed counts of true positives (TP), false positives (FP), and false negatives (FN). To ensure interpretability within the valid probability range, CI bounds falling outside the interval [0%, 100%] are truncated accordingly.

## Results

### Generated initial and refined dialogues

**Figure 8.**
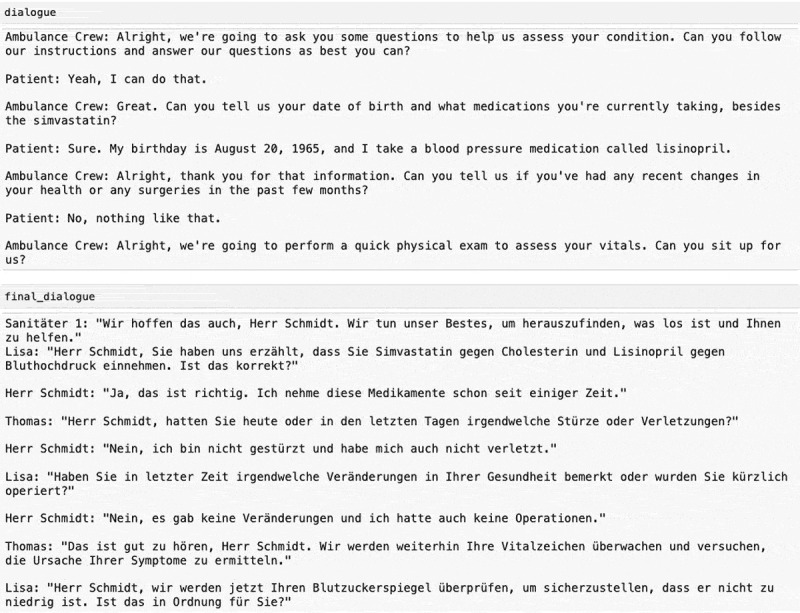
Comparison of English and German ambulance dialogue translations, showcasing the patient’s responses and the paramedic’s inquiries.

The data generation pipeline produced a total of 100 dialogues. The pipeline required several hours to process these 100 dialogues when run overnight. It should be noted that the execution time depends on the hardware used for inference with local models and the response times of OpenAI API services during execution, both of which may vary if reproduced. The initial English dialogues have an average length of 2,000 tokens, whereas the corresponding German dialogues have an average token length of approximately 4,000 tokens. The variation in the German dialogues is notably greater, with some dialogues extending beyond 10,000 tokens in the final corpus. One was excluded due to generated content deviating from meaningful interaction, becoming trapped in repetitive loops such as “Are you really okay?” and “Yes, I am really okay.” An example of an included dialogue is provided in Figure 8, illustrating a structured conversation in which a patient reports symptoms, allergies, and the events leading to the emergency, while paramedics conduct an assessment through targeted questioning.

Across *n* = 99 paired dialogues, the proportion of sentences labeled as positive in sentiment significantly decreased from 66.7% in the original dataset to 58.8% following the refinement step. A one-sided Wilcoxon signed-rank test confirmed this reduction as highly significant (W = 354, *p* ≈ 6.7 × 10^− 1 4^), suggesting that the refinement process does not introduce an artificial positivity bias. This is a critical finding, as retaining negatively toned expressions – such as distress or suicidal ideation – is essential for accurate medical information extraction. In the dual-judge evaluation, the expert’s composite ratings averaged 2.81 ± 0.19 (95% CI: 2.72–2.90), closely aligned with the LLM’s ratings of 2.86 ± 0.15 (95% CI: 2.78–2.93). A paired Wilcoxon test showed no significant difference between the two series (W = 36.0, *p* = .293), and inter-rater agreement across 152 item-level scores was fair (weighted Cohen’s κ = 0.30, 95% CI: 0.05–0.54). These results indicate that the LLM’s assessments approximate expert-level judgment and that refined dialogues consistently fall within the upper tier of the three-point quality scale. However, qualitative feedback from the human evaluator highlighted notable stylistic artifacts in the German dialogues, including redundant speech patterns and an overly formal register, which diverge from the fragmented, often elliptical nature of real emergency interactions. While not detrimental to coherence or clinical plausibility, such features may complicate downstream tasks such as fine-grained information extraction and somewhat limit conversational realism.

### Performance of RAG-based extraction on initial dialogues

The performance of the RAG-based text is summarized in Table 2, which demonstrates high accuracy across both nominal and numerical features. For nominal features, “Diagnosis” achieved high precision (86.87%) and perfect recall (100%), resulting in an F1-score of 92.97%. “Chief Complaint” showed slightly lower precision at 76.53%, but with high recall (98.68%), it reached an F1-score of 86.21%. “Medication Patient Reported” and “Medication Administered” both exhibited excellent precision (97.98% and 95.96%, respectively) and perfect recall (100%), leading to F1-scores of 98.98% and 97.94%. This indicates that the RAG-based extraction was especially effective in identifying medications correctly.

**Table 2.** Performance metrics of RAG-Based text extraction on the initial dialogues. Any 95% CI bounds below 0% or above 100% were clipped to the valid range [0%,100%].

	Ground truth feature	n	Precision initial, %	Recall initial, %	F1-score Initial, %	F1-score initial, 95% CI, %
Nominal						
	Diagnosis	99	86.87	100.00	92.97	(89.16–96.78)
	Chief complaint	99	76.53	98.68	86.21	(80.80–91.62)
	Medication patient reported	99	97.98	100.00	98.98	(97.57–100.00)
	Medication administered	99	95.96	100.00	97.94	(95.92–99.96)
Numerical						
	Blood Pressure	396	98.74	100.00	99.36	(98.81–99.92)
	Heart rate	198	98.48	100.00	99.24	(98.37–100.00)
	O2 saturation	198	93.43	100.00	96.61	(94.76–98.45)
	Respiration rate	198	100.00	100.00	100.00	(100.00–100.00)
	Temperature	198	100.00	100.00	100.00	(100.00–100.00)
	Pain score	99	100.00	100.00	100.00	(100.00–100.00)

For numerical features, “Blood Pressure” and “Heart Rate” exhibited near-perfect precision, with values of 98.74% and 98.48%, respectively, and achieved perfect recall of 100%, resulting in F1-scores of 99.36% and 99.24%. “O2 Saturation” demonstrated slightly lower precision at 93.43% but maintained perfect recall (100%), yielding an F1-score of 96.61%. “Respiration Rate,” “Temperature,” and “Pain Score” all achieved perfect precision, recall, and F1-scores of 100%, reflecting flawless extraction performance. This indicates that numerical values, compared to nominal features, posed minimal challenges for extraction.

Overall, the system demonstrates exceptional performance, particularly with numerical features, achieving near perfect or perfect accuracy. It also performs robustly with nominal features, although there is some variability in precision. However, the RAG-based system faces challenges in accurately identifying and extracting medical conditions, especially with the “Chief Complaint” feature. The lower precision in this area indicates a higher propensity for false positives and less accurate recognition of these critical features.

### Performance of RAG-based extraction on refined dialogues

The RAG-based text extraction system also performed well on refined dialogues, particularly excelling in recall across both nominal and numerical data categories, as shown in Table 3. However, compared to the initial dialogues, there was a noticeable decline in performance across several features, particularly in the Precision metric.

**Table 3.** Performance of RAG-based extraction on refined dialogues. Any 95% CI bounds below 0% or above 100% were clipped to the valid range [0%, 100%].

	Ground truth data	n	Precision refined, %	Recall refined, %	F1-score refined, %	F1-score refined, 95% CI, %
Nominal						
	Diagnosis	97	60.82	100.00	75.64	(68.13–83.15)
	Chief complaint	97	79.38	100.00	88.51	(83.51–93.51)
	Medication patient reported	97	94.74	97.83	96.26	(93.53–98.99)
	Medication administered	97	90.62	98.86	94.57	(91.24–97.90)
Numerical						
	Blood Pressure	388	72.16	100.00	83.83	(80.82–86.84)
	Heart rate	194	73.20	100.00	84.52	(80.37–88.68)
	O2 saturation	194	76.56	98.66	86.22	(82.35–90.08)
	Respiration rate	194	71.13	100.00	83.13	(78.78–87.49)
	Temperature	194	86.70	96.45	91.32	(88.35–94.28)
	Pain score	97	57.61	91.38	70.67	(62.77–78.57)

For nominal data, the extraction of “Diagnosis” had moderate precision at 60.82% (compared to 86.87% in the initial dialogues), but a perfect recall of 100% resulted in an F1-score of 75.64%. The “Chief Complaint” feature showed higher precision at 79.38%, with the same perfect recall, leading to a strong F1-score of 88.51%. The system was particularly effective at extracting “Medication Patient Reported,” achieving very high precision (94.74%) and recall (97.83%), resulting in an F1-score of 96.26%. Similarly, for “Medication Administered,” the system achieved high precision (90.62%) and near-perfect recall (98.86%), yielding an F1-score of 94.57%.

In the numerical data categories, the system maintained solid performance. “Blood Pressure” extraction had a precision of 72.16% with perfect recall, leading to an F1-score of 83.83%. “Heart Rate” extraction was similar, with 73.20% precision and 100% recall, resulting in an F1-score of 84.52%. For “O2 Saturation,” the system achieved a slightly higher precision of 76.56% and near-perfect recall (98.66%), leading to an F1-score of 86.22%. “Respiration Rate” showed a precision of 71.13% and perfect recall, yielding an F1-score of 83.13%. “Temperature” extraction had the highest precision in this group at 86.70%, with a recall of 96.45%, resulting in a strong F1-score of 91.32%. The “Pain Score,” while showing low precision at 57.61% (compared to 100% in the initial dialogues), benefited from a high recall of 91.38%, producing an F1-score of 70.67%.

Overall, the results indicate that the RAG-based system is particularly effective in recall, ensuring that the most relevant information is captured, with strong precision in several categories, leading to high F1-scores in most areas. However, it struggled, particularly in critical areas such as medical conditions “Diagnosis” and “Chief complaint” and numerical “Pain score” values, suggesting a need for further investigation and refinement to improve either the dialogue quality or RAG.

## Discussion

### Principal results

The experimental outcomes reveal distinct differences in the performance of the RAG-based extraction system between the initial and refined dialogues. Initially, the system exhibited exceptional accuracy, particularly with numerical features, achieving near-perfect precision and recall, resulting in F1-scores close to 100%. Nominal features also showed strong performance, although with some variability in precision, especially in the “Chief Complaint” category, where precision was somewhat lower. These results suggest that the system is highly effective at handling structured numerical data but encounters challenges when dealing with more complex nominal data that require nuanced interpretation, such as medical conditions.

When applied to the refined dialogues, the system’s performance declined, particularly in precision across both nominal and numerical categories. Despite maintaining high recall, the reduced precision indicates an increased rate of false positives. This decline in precision is particularly concerning in critical categories like “Diagnosis” and “Chief Complaint,” where accurate identification is essential for patient care. Contrary to our initial expectation that LLMs would render the conversations more positive, sentiment analysis revealed no such shift after refinement, so sentiment cannot explain the performance decline. Instead, precision appears to have suffered because the hierarchical-expansion workflow’s chained summarization steps removed clinically important details despite explicit retention prompts, and because the dialogue had to be translated into German for refinement and then back into English for extraction, during which categories such as “Diagnosis” and “Chief Complaint” may have been lost in the process. Taken together, these results point to two likely causes of the accuracy drop – first, that naturalizing the dialogue can itself hamper extraction, and second, that the subsequent back-translation into English further erodes the fidelity of critical clinical information.

Our dialogues achieve favorable results in a small-scale dual-judge evaluation. However, qualitative feedback from domain experts highlights persistent stylistic and pragmatic deficiencies. Notably, several dialogues exhibit what can be characterized as discourse-level hallucinations, such as over-generation and redundancy. These include echoic exchanges and excessively explicit verbalizations of standard procedures that, in practice, are rarely articulated. Such artifacts likely arise from the system’s two-stage prompting strategy, which (a) interjects intermediate summaries of prior turns and (b) mandates verbatim retention of all medical details across utterances. While these outputs remain factually accurate, they compromise the naturalness, coherence, and face validity of the interactions, potentially impairing usability in realistic clinical settings – particularly high-stakes environments like emergency care. The underlying issue is not factual hallucination per se but a breakdown in conversational grounding and pragmatic coherence. Addressing these limitations will require further refinement of generation mechanisms, potentially through the integration of domain-specific discourse constraints that better model clinical communication norms.

The quality of the generated dialogues plays a significant role in the performance of the RAG-based system. The refined dialogues, despite their intended improvements, introduced challenges that impacted the system’s precision. The initial dialogues, while more artificial and perhaps overly formal, provided a clearer structure for the system to extract relevant information. In contrast, the refined dialogues, which were adjusted to be more naturalistic, resulted in increased variability and less consistency in key features. This variability likely contributed to the observed decline in precision, as the system struggled to maintain the same level of accuracy when processing dialogues that more closely resemble real-life conversations.

Such a precision issue may be mitigated in practice through the integration of multimodal data sources within our RAG framework. Beyond textual retrieval, incorporating physiological signal interpretation – such as electrocardiography (ECG), oxygen saturation (SpO₂), and heart rate variability – could enhance contextual grounding and reduce reliance on verbal elaboration alone. These modalities provide situational context, improving the relevance and coherence of outputs in dynamic clinical environments. While body-worn cameras have been proposed as an additional data source (Ho et al. 2017), their use introduces substantial privacy risks. Given the sensitivity of audiovisual recordings in medical contexts, we recommend against their deployment in favor of privacy-preserving alternatives such as ambient sensors, wearable biomedical devices, or secure, selectively anonymized data streams that comply with clinical data governance standards.

### Comparison with prior work

When compared to existing methods, especially BERT-based models for text extraction (Liu et al. 2021; Ramos et al. 2024; Zhu, Tu, and Huang 2021), the RAG-based system exhibits both notable strengths and weaknesses. One of its significant advantages lies in its ability to achieve near-perfect performance with numerical features, particularly in the more structured initial dialogues. This is a critical strength, as many existing methods struggle with accurately extracting numerical data from unstructured text. However, a key weakness of the RAG-based system becomes evident as its precision declines in more refined and complex dialogues, which often feature less structured language. In contrast, existing methods, such as fine-tuned encoder models (Harnoune et al. 2021; Rasmy et al. 2021), though potentially less effective in recall, often maintain more consistent precision. The challenge with these methods is the need to fine-tune a separate encoder model for each feature type, which still may not comprehensively address all types of data, making these methods less practical for widespread clinical application

It is important to emphasize that our relatively good results were achieved without the use of fine-tuning. Unlike Tang et al. (2023), we do not assume that additional fine-tuning with medical data is necessary to reach acceptable solutions when models are sufficiently large (≥7B). The survey by Shi et al. (2024) further supports this, indicating that prompt-based approaches with existing LLMs have been predominantly successful. This is not to say that we do not recognize the potential for improvements through fine-tuning. Dong et al. (Yuan et al. 2024) have demonstrated that a continuously pre-trained automatic medical note generation system using a 13B LLaMA2 model can outperform GPT-4. However, a significant bottleneck lies in acquiring a sufficient volume of high-quality data. Moreover, even when the data is available, substantial computational resources are required, which many institutions may find prohibitive. Additionally, such models often lose the capacity for general reasoning, making them functionally closer to encoder-based architectures.

Recent work by Nori et al. (2023) reinforces our position by showing that generalist foundation models, when paired with systematic prompt engineering strategies such as Medprompt, can outperform highly specialized models like Med-PaLM 2 across diverse medical benchmarks. Notably, this was achieved without task-specific fine-tuning and with significantly fewer model calls. These findings highlight the value of decoder-based, general-purpose LLMs augmented with advanced prompting techniques and RAG, especially in resource-constrained settings. This strategy leverages broad generalization capabilities while avoiding the rigidity and infrastructure demands associated with task-specific fine-tuning.

It is important to emphasize that our relatively good results were achieved without the use of fine-tuning. Unlike Tang et al. (2023), we do not assume that additional fine-tuning with medical data is necessary to reach acceptable solutions when models are sufficiently large (≥7B). The survey by Shi et al. (2024) further supports this, indicating that prompt-based approaches with existing LLMs have been predominantly successful. This is not to say that we do not recognize the potential for improvements through fine-tuning. Dong et al. (Yuan et al. 2024) have demonstrated that a continuously pre-trained automatic medical note generation using a 13B LLaMA2 model can outperform GPT-4. However, a significant bottleneck lies in acquiring a sufficient volume of high-quality data for such fine-tuning. Moreover, even when the data is available, fine-tuning requires substantial computational resources, which many institutions may find prohibitive. Additionally, fine-tuned models often lose the capacity for general reasoning, making them more akin to encoder model. Therefore, we recommend employing sophisticated prompting techniques, particularly in combination with RAG when using offline LLMs. This approach leverages the existing capabilities of LLMs while mitigating the resource-intensive demands of fine-tuning.

### Limitations

The observed decline in precision, particularly in critical nominal entities such as Diagnosis and Chief Complaint, poses a substantial risk that transcends technical inaccuracies. These elements are central to clinical reasoning, guiding diagnostic trajectories, therapeutic interventions, and overall patient management. Errors in their extraction can propagate through the clinical workflow, leading to misdiagnoses, suboptimal or delayed treatments, and compromised patient safety. The implications are especially severe in high-acuity settings – such as emergency or critical care – where rapid decisions must be made under tight temporal constraints. In such contexts, even minor inaccuracies can have disproportionate consequences. Mitigating this risk necessitates robust human oversight at key decision points, emphasizing that precision in these core features is not merely a technical objective but a clinical imperative.

Although the system targets German-language clinical use, the evaluation of the final extraction performance was conducted on back-translated English texts due to the lack of sufficiently annotated German emergency datasets. However, we acknowledge that this introduces potential confounding variables related to translation fidelity and linguistic nuance (Follesdall 2000). For instance, German trade names of medications often differ significantly from their English counterparts, which may complicate maintaining the contextual link to the associated medical condition. To mitigate these effects, all translations were carefully reviewed for clinical accuracy, and the synthetic dialogues were originally generated and processed in German prior to translation. We recognize that native-language performance may differ, particularly in the handling of idiomatic expressions, abbreviations, and domain-specific terminology. Future work will prioritize large-scale evaluation directly on German-native clinical texts, ideally with real-world data from emergency departments, to fully assess the system’s native-language robustness and to reduce the reliance on translated evaluation pipelines.

Assuming perfect performance from speech-to-text (STT) systems overlooks critical limitations, particularly in emergency medicine, where speech may be slurred, whispered, unclear, or entirely absent. These constraints necessitate a multimodal approach to data acquisition and communication. Optimizing microphone placement and hardware specifications is essential to maximize audio quality. Moreover, integrating complementary data sources – such as vital signs from monitors – can contextualize and compensate for deficient verbal input. Clinicians should be encouraged to adopt a deliberately verbose communication style, including verbalizing actions, repeating key information, and providing descriptive commentary. Such practices enhance STT reliability and improve shared situational awareness. Environmental factors, including high background noise, further complicate transcription accuracy, highlighting the need for real-time feedback mechanisms that alert users to potential errors in speech capture and transcription.

## Conclusions

The system exhibited near-perfect accuracy in processing numerical features; however, it encountered significant challenges with nominal features, particularly in the “Diagnosis” and “Chief Complaint” categories, where precision was markedly reduced. This decrease in precision, especially in dialogues designed to closely emulate real-world clinical interactions, raises critical concerns regarding the system’s reliability in accurately extracting essential medical information. These findings highlight the system’s robustness in handling structured numerical data while simultaneously exposing its vulnerabilities in processing the complex and nuanced language necessary for accurate clinical decision-making. This discrepancy underscores the need for further refinement in the system’s linguistic capabilities to ensure its effectiveness in real-world healthcare settings.

To address the limitations identified, future research should prioritize several key areas. Firstly, refining dialogue generation processes to ensure more naturalistic conversations is essential. This can be achieved by fine-tuning models using domain-specific medical texts, particularly in underrepresented languages such as German. Additionally, advancements in underlying extraction algorithms, especially for nominal features, are crucial to improving precision and reducing the incidence of false positives. Another promising avenue for exploration is the comprehensive integration of multimodal data sources, such as outputs from medical devices, to complement and enhance the accuracy of speech-to-text systems in critical care settings. Moreover, iterative refinements in the performance of rapidly evolving LLMs and RAG techniques, supported by collaborative research initiatives, will be pivotal in ensuring their successful application in healthcare. These advancements are essential for reliably supporting clinical decision-making and ultimately improving patient outcomes.

## Data Availability

The data utilized in this study is derived from the MIMIC-IV dataset, a publicly available resource hosted on PhysioNet and provided by the Massachusetts Institute of Technology. The dataset consists of de-identified patient data from Beth Israel Deaconess Medical Center in Boston, designed for research applications in healthcare and biomedical fields. The specific subset used in this study, MIMIC-IV-ED, focuses on Emergency Department (ED) encounters, offering comprehensive records including patient demographics, diagnoses, vital signs, and treatments. The MIMIC-IV dataset can be accessed through PhysioNet at https://physionet.org/content/mimiciv/2.2/under the appropriate data use agreement. As per the Taylor & Francis Open Data Policy, we affirm that the data supporting this study is openly available through PhysioNet. Any further details or modifications applied to the dataset for this study is available at https://github.com/denMo24/AIDoCS/. Researchers seeking access to MIMIC-IV data must complete the required training and obtain approval through PhysioNet’s application process.
